# Evaluation of the Anti-Inflammatory Pain Effect of Ginsenoside-Conjugated O-Carboxymethyl Chitosan Particles

**DOI:** 10.3390/polym15194011

**Published:** 2023-10-06

**Authors:** Huan-Jun Lu, Jian-Ke Cen, Yu Ren, Mei-Xian Li

**Affiliations:** 1 Institute of Pain Medicine and Special Environmental Medicine, Nantong University, Nantong 226019, China; huanjunlu@ntu.edu.cn (H.-J.L.); cenjianke@outlook.com (J.-K.C.); 2 National and Local Joint Engineering Research Center of Technical Fiber Composites for Safety and Protection, School of Textile and Clothing, Nantong University, Nantong 226019, China

**Keywords:** O-carboxymethyl chitosan, ginsenoside Rh2, anti-nociception, inflammatory pain

## Abstract

Nanoparticle delivery of functional molecules or vaccines is an effective method for the treatment of many diseases. This study aims to design ginsenoside Rh2-conjugated O-carboxymethyl chitosan (O-CMC/Rh2) as a drug delivery system and explore its anti-nociceptive effects. O-CMC/Rh2 was synthesized with an esterification reaction, and its chemical composition and morphology were evaluated using proton nuclear magnetic resonance (^1^H NMR), the attenuated total reflectance-Fourier transform infrared (ATR-FTIR) spectroscopy, and scanning electron microscopy (SEM). In addition, the in vitro cumulative release of Rh2 from the O-CMC/Rh2 was also evaluated under different pH conditions. The results showed that the ginsenoside Rh2 was successfully conjugated to the O-CMC matrix and exhibited a highly porous structure after conjugation, facilitating the release of Rh2 from O-CMC. Complete Freund’s adjuvant (CFA) and burn injury-induced pain models were used to evaluate the anti-nociceptive effects of O-CMC/Rh2 on inflammatory pain. O-CMC/Rh2 reduced CFA-induced pain hypersensitivity in a dose-dependent manner and had a longer analgesic effect than Rh2. In addition, O-CMC/Rh2 also relieved the chronic pain induced by bury injury. These results indicated that O-CMC/Rh2 could be useful in reducing inflammatory pain, thus possessing a potential medicinal application in pain therapy.

## 1. Introduction

Pain is the most common clinical symptom. These symptoms are often accompanied by insomnia, anxiety, and other mental disorders [1]. Although the pain may not be fatal, it seriously affects the patients’ normal physiological functions and quality of life. Approximately 30% of adults experience chronic pain [2]. Two out of three outpatients have symptoms of various types of pain, and there are at least 400 million patients with pain in China [3]. Inflammatory pain is a common chronic pain (e.g., joint pain, neuropathic pain, and burning pain). Unlike acute pain, chronic pain generally lasts for a long time and has a complex mechanism [4,5,6]. Clinical medications for chronic pain can be divided into opioids (cocaine and morphine) and non-steroidal anti-inflammatory drugs. Although opioids have good analgesic effects, they carry a significant risk of severe side effects, including itching, addition, tolerance, and dependence [7,8]. Therefore, exploring new types of analgesic drugs with minimal adverse reactions is a direction for future research. Ginsenosides, which are also known as triterpene saponins, are steroid compounds and are mainly found in the traditional herbal plant ginseng (Panax ginseng C.A. Meyer). Nearly 200 types of ginsenosides have been identified in ginseng, including Rb1, Rb2, Rg1, Rd, Rh1, and Rh2. Increasing clinical and experimental evidence indicates that ginsenosides have different advantageous effects on various diseases [9,10,11,12]. Ginsenoside Rh2 is a major bioactive component of red ginseng extract. Recent studies have reported that Ginsenoside Rh2 has various pharmacological effects, such as antidepression [13], antipruritic [14], tumor growth inhibition [15] and anti-viral activity [16], making it a potential anticancer drug. Although ginsenoside Rh2 has various biological effects, its efficacy and safety may depend on its dosage and diffusion rate. Therefore, ginsenoside Rh2 has been explored for its potential application in drug release systems such as encapsulated nanoparticles, three-dimensional network structure hydrogels, encapsulated microspheres, improving their stability and bioavailability and providing sustained release and targeted delivery. In addition, Rh2 is a small, lipid-soluble molecule with a fast diffusion rate and poor water solubility. Thus, in order to enhance the biological efficacy of Rh2 within the body, it is imperative to increase its solubility, extend its duration of action within organisms, and regulate its rate of release. Rh2 can be conjugated with some water-soluble biomaterials or incorporated into liposomes which are lipid-based vesicles that can encapsulate drugs to improve their solubility and stability, as well as control their release kinetics. However, research on the Rh2 compound is still relatively limited and still requires further research to optimize their effectiveness and safety.

Chitosan, as one of the most popular polysaccharide biopolymers, which is deacetylated from chitin with cationic properties, has been widely used as promising carriers of therapeutic agents for drug delivery systems because of its nontoxicity, biocompatibility, biodegradability, antimicrobial activity, low immunogenicity, low cost, and so on [17,18]. In addition, the drug-loaded chitosan system can protect drugs from degradation, enhance drug stability, and provide controlled release profiles to improve drug delivery efficiency. Moreover, chitosan enhanced the epithelial permeability by disrupting intercellular tight junctions [19]. However, chitosan has a poor solubility in water and can only be dissolved in acidic solvents because of its stable crystalline structure, thus it has limited applications in biomedicine. To solve this problem, water-soluble chitosan derivatives, such as O-carboxymethyl chitosan (O-CMC), N,O-carboxymethyl chitosan (N,O-carboxymethyl chitosan), (N,N-carboxymethyl chitosan), and catechol-conjugated chitosan have been developed by the chemical modification, retaining the unique properties of chitosan [20,21]. Among these derivatives, O-carboxymethyl chitosan (O-CMC) is a modified form of chitosan where carboxymethyl groups are introduced onto the chitosan backbone to enhance the water solubility and to introduce new functional groups to chitosan, expanding its potential applications. The water solubility of O-CMC allows for the encapsulation of hydrophobic drugs, and its functional carboxymethyl groups can facilitate drug loading and release. In addition, O-CMC has many outstanding properties including antimicrobial properties, anticancer properties, antioxidant activities, and excellent pH sensitivity. For these advantages, it is widely used in various biomedical applications including drug delivery, wound healing, tissue engineering, and so on. Although O-CMC has shown promising results in various areas, further research is still needed to fully understand its mechanisms of action, optimize its properties, and evaluate its efficacy and safety in clinical applications, which may vary depending on the intended use and target organism or cells.

In the present study, we prepared a drug delivery system through ester bond conjugation of the ginsenoside Rh2 with water-soluble O-CMC to release Rh2 in a controlled manner, improving the drug solubility and release stability. The physiochemical characteristics of the O-CMC/Rh2 were analyzed using proton nuclear magnetic resonance (^1^H NMR), and attenuated total reflectance-Fourier transform infrared spectroscopy (ATR-FTIR), and its morphology was evaluated using scanning electron microscopy (SEM). In addition, the in vitro cumulative release of Rh2 from the O-CMC/Rh2 was also studied under different pH conditions (pH 7.4 and pH 5.8). We found that Rh2 was successfully conjugated to the O-CMC, and the obtained O-CMC/Rh2 was a pH-dependent sustained release matrix. The cumulative Rh2 release from O-CMC/Rh2 at pH 5.8 was higher than at pH 7.4, and the release rate at pH 5.8 was faster at the early stage, whereas that at pH 7.4 became faster up to 40 h. Furthermore, the anti-nociceptive effects of O-CMC/Rh2 on CFA and burning-induced inflammatory pain were evaluated. We found that O-CMC/Rh2 attenuated inflammation-induced mechanical allodia and showed a longer analgesic effect than Rh2 alone. This study provides a promising drug delivery system for improving the solubility of insoluble drugs, controlling their release rates, and prolonging analgesic effect.

## 2. Materials and Methods

### 2.1. Materials

O-carboxymethyl chitosan (O-CMC) was purchased from Qingdao Honghai Bio-Technology Co., Ltd. (Qingdao, China). N,N′-dicyclohexylcarbodiimide (DCC) was provided by Solarbio (Beijing, China), and ginsenoside Rh2 was purchased from Feiyu Bio (Nantong, China). Methanol was provided by Aladdin (Shanghai, China), and 4-dimethylaminepyridine (DMAP) was obtained from Macklin (Shanghai, China). All chemicals were used as received without further purification.

### 2.2. Synthesis of Ginsenoside Rh2-Conjugated O-Carboxymethyl Chitosan (O-CMC/Rh2)

0.1 mmol of O-CMC was dissolved in 25 mL of distilled water and stirred using a magnetic stirrer until completely dissolved, and then 0.15 mmol of DCC and 0.3 mmol of DMAP were added to the O-CMC solution while stirring for 20 min. After that, Rh2 solution (0.1 mmol in 1 mL methanol) was added while stirring continuously for 24 h at room temperature and then dialyzed against distilled water for 24 h using a dialysis membrane (MWCO = 3500 kDa). Finally, O-CMC/Rh2 solution was freeze-dried at −40 °C for 48 h to obtain O-CMC/Rh2 conjugates with a yield of 73.4%.

### 2.3. Characterization of O-CMC/Rh2

The chemical compositions of O-CMC and O-CMC/Rh2 were determined using proton nuclear magnetic resonance (^1^H-NMR, AVANCE III HD 400, Bruker, Fällanden, Switzerland). Both the O-CMC and O-CMC/Rh2 matrix were dissolved in D_2_O at a concentration of 1.0 *w*/*v*%. In addition, the degree of Rh2 substitution (DS) was calculated through the ^1^H-NMR spectra.

Furthermore, the attenuated total reflectance (ATR)-FTIR spectra of O-CMC and O-CMC/Rh2 were recorded using Nicolet IS 50 spectrometers (Thermo Scientific, Waltham, MA, USA) over a frequency range of 500–4000 cm^−1^ to confirm the successful synthesis of O-CMC/Rh2.

Finally, the morphology was investigated by field-emission scanning electron microscopy (FESEM, ZEISS Gemini SEM 300, Oberkochen, Germany). The obtained matrix was sputter-coated with gold in an argon atmosphere, and SEM analysis was performed at an accelerating voltage of 5 kV.

### 2.4. In Vitro Cumulative Release of Rh2 Form O-CMC/Rh2

In vitro cumulative release of Rh2 from the O-CMC/Rh2 matrix was analyzed using a UV spectrophotometer at a wavelength of 203 nm. Briefly, O-CMC/Rh2 matrices (3 mg/mL) were dispersed in distilled water (1 mL), which was then transferred to a dialysis membrane tubes (MWCO: 3500), followed by being placed in a PBS buffer (pH 7.4) or acetate buffer (pH 5.8). It was then gently shaken at 37 °C at 100 rpm. At predetermined time intervals (20, 40, 60, and 80 h), a 1 mL incubation medium was withdrawn and replaced with fresh medium.

### 2.5. Animals

Adult male C57/B6 mice (8 weeks, weighing 26–30 g) were obtained from the Experimental Animal Center of Nantong University. Animals were housed in a 12:12 light-dark cycle and temperature-controlled (room temperature around 22 ± 1 °C) environment with free access to food and water. About 6–7 mice were housed in a cage and randomly segregated into several groups for each experiment. All the mouse experiments were performed according to ethics protocols approved by the Animal Care and Use Committee of Nantong University. All animal treatments were performed in accordance with the guidelines of the International Association for the Study of Pain. The experimenters were blinded to the treatment during the experiments.

### 2.6. CFA-Induced Inflammatory Pain Model

The CFA-induced inflammatory pain model was established as described previously [22]. After mice were anesthetized with isoflurane (2%, RWD Life science, San Diego, CA, USA), 20 μL CFA (100%; Sigma-Aldrich, St. Louis, MI, USA) was injected into the mice’s paws to induce inflammatory pain. Control mice were injected with the same amount of saline in their paws. The nanoparticles were dissolved in saline and intrathecally injected into the L5 and L6 spinal cord intervertebral spaces to deliver the reagents to cerebrospinal fluid. All groups of mice were allowed to acclimatize to the home cage and environment. After the CFA injection, all groups of mice underwent a nociceptive behavior test (von Frey Hair test and Hargreaves test) for 72 h.

### 2.7. Burn Injury Induced Inflammatory Pain Model

A burn injury-induced pain model was established as described previously [23]. Briefly, after the animals were anesthetized with isoflurane (2%, RWD Life science) and maintained for several minutes before the procedure and continuously through the du-ration of the experiments. Then, the right hind paws of the mice were placed in contact with a 65 °C-heating plate for 15 s. For the sham surgery, the right hind paws were placed in contact with a room-temperature heating plate for 15 s. After the heating plate exposure, the template was removed, and the animals were allowed to recover from anesthesia. The nociceptive responses (Mechanical allodynia and thermal hyperalgesia) of the mice were assessed using the von Frey and Hargreaves test.

### 2.8. Von Frey Hair Test

The mechanical sensitivity of the mice was measured using the von Frey test as previously described [24]. Briefly, the animals were placed on an elevated metal mesh floor and habituated for 30 min before the experiments. The plantar surface of the hind paw was stimulated using a series of von Frey hairs with logarithmically incrementing stiffness (0.02–2.56 g; Stoelting). Withdrawal was considered a response to the stimulus of von Frey hair. The 50% paw-withdrawal threshold was determined using Dixon’s up-down method.

### 2.9. Hargreaves Test

The Hargreaves test was used to quantify the thermal threshold of the mouse paws using radiant thermal stimulation [24]. Briefly, the animals were placed on a glass plate and allowed to habituate for 30 min before the experiments. The paw surface was exposed to radiant heat through a transparent glass surface until a withdrawal response was observed. The heat radiation intensity was set to obtain withdrawal latencies of approximately 10–14 s as the baseline. A maximum cut-off time of 20 s was set to prevent potential tissue injury. All behavioral experiments were performed by individuals who were blinded to the treatment.

### 2.10. Statistics

All results are shown as mean ± SEM values. Behavioral data were analyzed using one-way or two-way analysis of variance (ANOVA), and differences between two groups were compared using Student’s *t*-test. The criterion for statistical significance was set at *p* < 0.05.

## 3. Results and Discussion

### 3.1. Synthesis of O-CMC/Rh2

**Figure 1:**
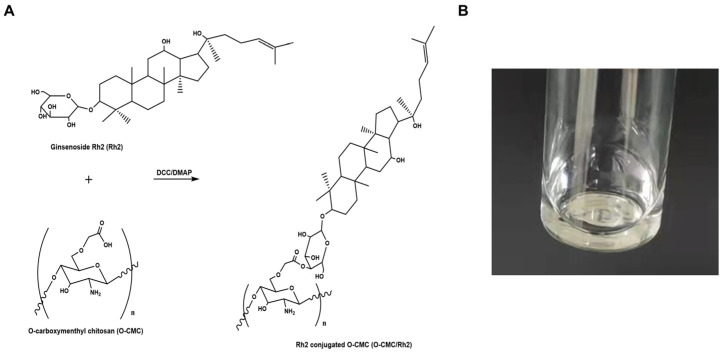
(**A**) Schematic of ginsenoside Rh2-conjugated O-carboxymethyl chitosan (O-CMC/Rh2), (**B**) image of the aqueous solution of O-CMC/Rh2.

As shown in Figure 1A, the chemical synthesis of O-CMC/Rh2 conjugates was completed via the formation of ester bonds between the −COOH group of O-CMC and the OH group of Rh2. In addition, as can be seen in Figure 1B, the prepared O-CMC/Rh2 could be dissolved in water at high concentrations, whereas free Rh2 is insoluble at the same concentration. This indicates that the conjugation of O-CMC could enhance the solubility of Rh2, which could enhance the efficacy of Rh2.

**Figure 2:**
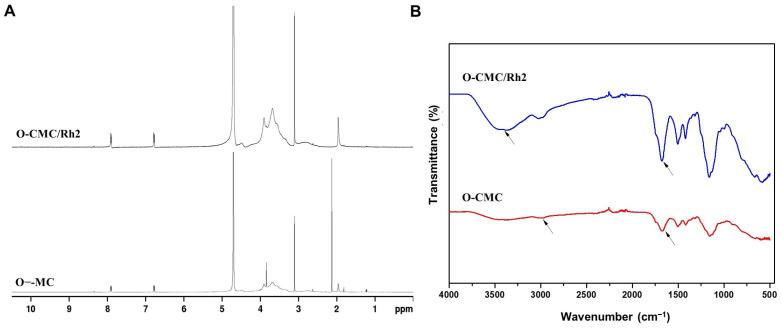
This (**A**) ^1^H NMR and (**B**) FTIR spectra of the O-CMC and O-CMC/Rh2 conjugates.

Figure 2A shows the ^1^H NMR spectra of O-CMC and O-CMC/Rh2. The D_2_O peak at 4.85 ppm was used as the reference peak. As expected, the ^1^H-NMR spectrum of O-CMC/Rh2 showed characteristic peaks corresponding to O-CMC and Rh2 (0–2 ppm [25]), exhibiting successful conjugation of O-CMC and Rh2. The degree of substitution (DS) of Rh2 can be calculated based on the integration ratio of the proton peak from the methyl group of Rh2 to that of the −OCH_2_=O− peak of O-CMC, showing the Rh2 conjugation with a DS of 4.98. 

### 3.2. Characterization of O-CMC/Rh2

An ATR-FTIR spectrum was employed to investigate the changes in chemical structure between O-CMC and O-CMC/Rh2. As shown in Figure 2B, the absorption peak of O-CMC at approximately 3400 cm^−1^ was attributed to the O-H stretching overlapping with the N-H stretching, while it, at approximately 2900 cm^−1^, was attributed to aliphatic C-H stretching [26]. In addition, the IR spectrum of O-CMC shows strong peaks at 1671 cm^−1^ and 1414 cm^−1^ corresponding to the asymmetric and symmetric strength vibration of the COO^−^ group, while the absorption peak at 1681 cm^−1^ for O-CMC/Rh2 was attributed to the C=O groups, which confirmed the esterification of O-CMC/Rh2. Compared to O-CMC, the intensities at approximately 1700 cm^−1^, 2900 cm^−1^, and 3420 cm^−1^ for O-CMC/Rh2 increased after esterification. It indicates that Rh2 was successfully conjugated to the O-CMC through ester bonding.

**Figure 3:**
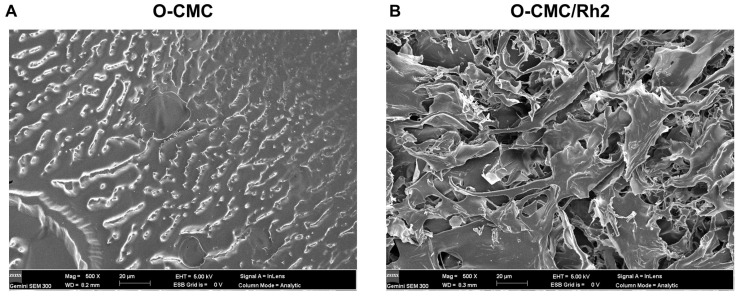
FE-SEM images of O-CMC (**A**) and O-CMC/Rh2 (**B**) captured at an accelerating voltage of 5 kV.

The surface morphologies of O-CMC and O-CMC/Rh2 were examined using SEM. The SEM images of O-CMC and O-CMC/Rh2 are shown in Figure 3. It can be seen that the surface morphology of O-CMC is a dense structure (Figure 3A), whereas that of O-CMC/Rh2 is a highly porous structure, indicating a better release of Rh2 from O-CMC (Figure 3B).

### 3.3. In Vitro Cumulative Release of Rh2 from O-CMC/Rh2

**Figure 4:**
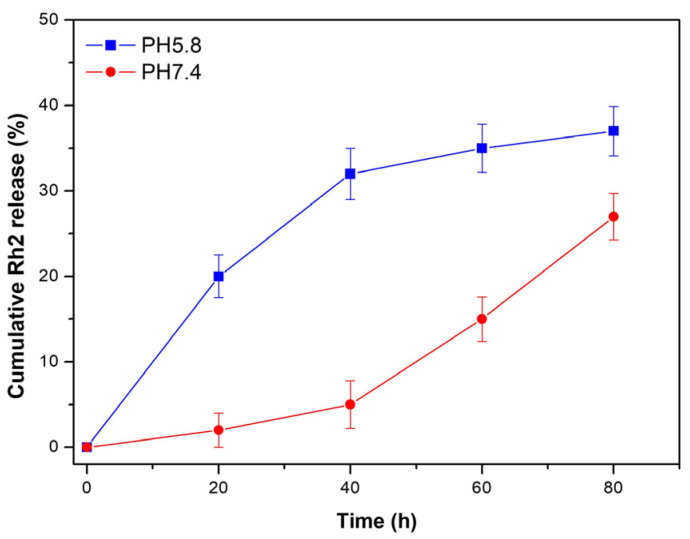
In vitro cumulative release of Rh2 from O-CMC/Rh2 matrix at pH 7.4 and pH 5.8.

The in vitro cumulative release profile from O-CMC/Rh2 at different pH buffers (pH 7.4 and pH 5.8) was investigated to evaluate its effectiveness in the drug delivery system. Generally, the in vivo environment becomes less acidic during an inflammatory infection, thus two pH conditions were chosen to compare its cumulative release profile. The cumulative Rh2 released from O-CMC/Rh2 matrix under physiological conditions (pH 7.4) and pathophysiological conditions (pH 5.8), as is shown in Figure 4. It can be seen that the release rate of Rh2 under pathophysiological conditions (pH 5.8) is faster at the early stage, and becomes slow at around 40 h. On the contrary, the release rate of Rh2 under physiological condition (pH 7.4) is relatively slow before 40 h, and it becomes faster at around 40 h. It may be that the ester bond between O-CMC and Rh2 can be easily broken in slightly acidic conditions, causing a large amount of Rh2 to release at the initial stage at a pH of 5.8. However, it takes much more time to break the ester bond under physiological conditions (pH 7.4), thus the release rate at the initial stage is slower than that up to 40 h. Figure 4 shows that the cumulative Rh2 release from O-CMC/Rh2 at pH 5.8 is higher than that at pH 7.4, which is consistent with the explanation described above. These results indicate that O-CMC/Rh2 is a pH-dependent sustained release matrix, providing a potential application as a drug delivery system of O-CMC/Rh2. 

### 3.4. Impact of O-CMC on CFA-Induced Inflammatory Pain

**Figure 5:**
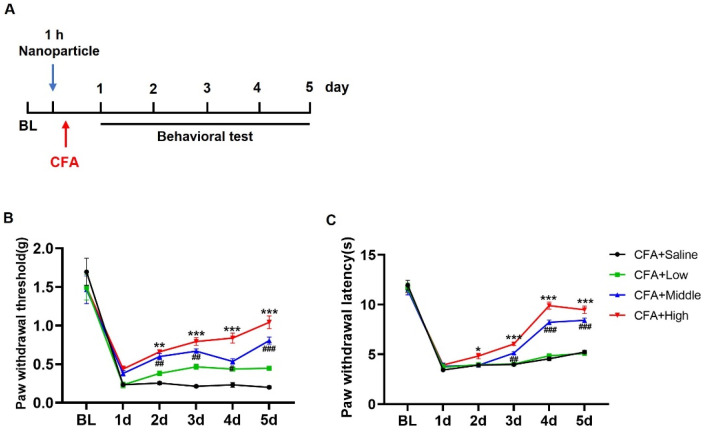
O-CMC/Rh2 attenuates CFA-induced paw mechanical allodynia and thermal hyperalgesia. (**A**) Schematic timeline for the administration of CFA injection, O-CMC/Rh2 treatment, and behavior detection. (**B**) Change in mechanical allodynia and (**C**) thermal hyperalgesia of CFA-injected mice treated with various doses of O-CMC/Rh2. Two-way analysis of variance (Two-way ANOVA) was employed. *n* = 6 mice/group. * *p* < 0.05, ** *p* < 0.01, *** *p* < 0.001, CFA vs. CFA + O-CMC/Rh2 (High), # *p* < 0.05, ## *p* < 0.01, ### *p* < 0.001, CFA vs. CFA + O-CMC/Rh2 (Middle).

The complete Freund’s adjuvant (CFA)-induced inflammatory pain model is a widely used method in preclinical research to study inflammatory pain. Thus, we chose the CFA-induced inflammatory pain model to compare the anti-nociceptive effects of O-CMC/Rh2 on the O-CMC/Rh2-treated mice and the control mice. Different doses of Rh2 were intrathecally injected 1 h before the CFA injection (Figure 5A). The development of mechanical hyperalgesia and thermal allodynia was evaluated for several days after CFA injection (Figure 5A) and the CFA-induced mechanical allodynia was evaluated from day one to five after the CFA injection (Figure 5B). The administration of O-CMC/Rh2 significantly attenuated mechanical allodynia at high (100 μM) and moderate (10 μM) doses of O-CMC/Rh2, but not at low doses (1 μM). Rh2 also attenuated CFA-induced thermal hyperalgesia from days two to five at high and moderate doses of O-CMC/Rh2, but not at low doses (Figure 5C). These data indicate that O-CMC/Rh2 administration relieve CFA-induced pain hypersensitivity in a dose-dependent manner.

### 3.5. O-CMC/Rh2 Has a Longer Analgesic Effect than Rh2

**Figure 6:**
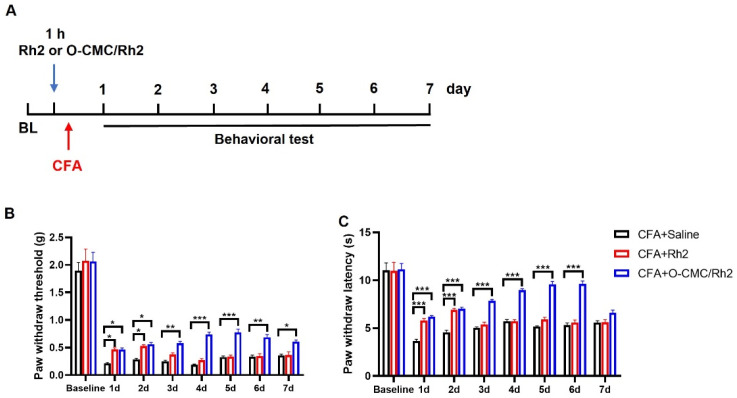
O-CMC/Rh2 has a longer analgesic effect compared to Rh2. (**A**) Experimental flowchart for intrathecal administration of Rh2 or O-CMC/Rh2 nanoparticle and behavior detection. (**B**) Mechanical allodynia threshold and thermal hyperalgesia latency (**C**) in CFA + Saline, CFA + Rh2 (10 μM), and CFA + O-CMC/Rh2Promotion of the genipin crosslinked chitosan-fiber (10 μM) groups are continuously measured for 7 d. Two-way analysis of variance (Two-way ANOVA) was employed. *n* = 6 mice/group. * *p* < 0.05, ** *p* < 0.01, *** *p* < 0.001.

Rh2 is a small lipid-soluble molecule with a high diffusion rate and low biological availability in the body [27]. We wanted to know whether O-CMC confusion could improve the biological availability of Rh2 in vivo. Therefore, we compared the analgesic durations of Rh2 and O-CMC/Rh2. As for the CFA injection-induced inflammatory pain, pain behavior was measured for seven consecutive days (Figure 6A). At d1 and d2 after injection, Rh2 (10 μM) and O-CMC/Rh2 (10 μM) significantly relieved the mechanical hyperalgesia and heat pain caused by inflammation in the mice (Figure 6B,C). The analgesic effect of Rh2 lasted for only 48 h; however, the relief effect of O-CMC/Rh2 on mechanical pain was maintained until d7, and the relief effect on thermal pain was maintained until d6 (Figure 6B,C). These results indicated that O-CMC/Rh2 had a longer analgesic effect in vivo than Rh2.

### 3.6. Impact of O-CMC/Rh2 on Burning Injury-Induced Inflammatory Pain

**Figure 7:**
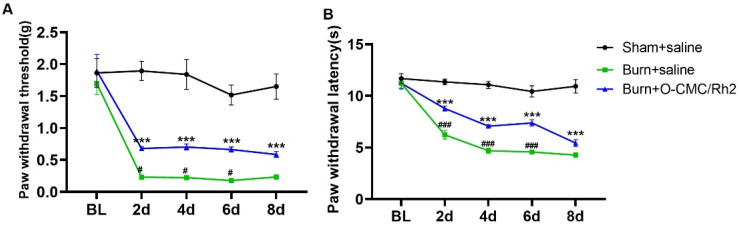
O-CMC/Rh2 attenuates burning injury-induced paw mechanical allodynia and thermal hyperalgesia. (**A**) Comparison of paw mechanical allodynia and (**B**) thermal hyperalgesia among Sham, burn + saline, and burn + O-CMC/Rh2 nanoparticle (10 μM) groups. Two-way analysis of variance (Two-way ANOVA) was employed. *n* = 6 mice/group. *** *p* < 0.001, Sham vs. burn + saline group, ### *p* < 0.001, burn + saline vs. burn + O-CMC/Rh2 nanoparticle group.

Similar to the CFA-induced inflammatory pain model, we also used another model of inflammatory pain (burning injury-induced pain) to verify the analgesic effects of O-CMC/Rh2. Burn injury-induced pain is a common type of inflammatory pain. When the skin is burned, the damaged tissue releases various inflammatory mediators, such as cytokines, chemokines and prostaglandins, and finally causes pain. We found that the mice showed a significant decrease in the paw withdrawal threshold (mechanical allodynia) after the hind-paw burn injury, which was until to 8th day. Meanwhile, the paw withdrawal latency (thermal hyperalgesia) of the burn + saline group mice also decreased compared to sham + saline group mice. However, compared to burn + saline group, the administration of O-CMC/Rh2 (10 μM) significantly attenuated burn injury-induced mechanical allodynia and hyperalgesia (Figure 7A,B). In addition, O-CMC/Rh2 showed the analgesic effects from the first behavior-testing day and also persisted for at least for one week (Figure 7A,B). These results showed that O-CMC/Rh2 exhibited beneficial analgesic effects against burning injury-induced pain.

## 4. Conclusions

Nanotechnology has revolutionized drug delivery by providing a platform for the development of targeted and controlled-release drug delivery systems. The unique properties of nanoparticles, such as their small size, large surface area, and ability to penetrate biological barriers, make them ideal candidates for drug delivery [28]. One of the most promising applications of nanotechnology in drug delivery is in the development of targeted drug delivery systems. Nanoparticles can be designed to specifically target cells or tissues, allowing for the delivery of drugs directly to the site of action. This can improve the efficacy of drugs while reducing side effects and toxicity to healthy cells [29]. Nanoparticles can be functionalized with molecules that specifically bind to target cells, such as antibodies or peptides, allowing for specific targeting. Nanoparticles can also be used for the delivery of poorly soluble drugs. Many drugs have low solubility in water, which can limit their efficacy and bioavailability. Nanoparticles can be used to solubilize these drugs, improving their delivery and efficacy. Nanoparticles can also protect drugs from degradation and clearance, allowing for prolonged drug activity [30]. As scaffolds, chitosan and its derivatives have been used to develop different types of drug delivery systems, including capsules [25,26], hydrogels [31,32], and membranes [33,34]. It has been reported that drugs conjugated with polymers exhibit prolonged half-life, higher stability and water solubility, as well as lower immunogenicity [35]. In this study, we developed a water-soluble Rh2-conjugated O-CMC for the stable release of Rh2, and the results indicated that O-CMC/Rh2 was more effective in relieving inflammatory pain and prolonging the duration of pain relief than Rh2. Rh2 was conjugated to O-CMC through an esterification reaction, thus Rh2 was released by breaking the ester bond. Moreover, the obtained O-CMC/Rh2 was pH-sensitive, controlling the Rh2 release rate according to pH, which changed at the site of injury during inflammatory pain. The results show that the release rate of Rh2 for O-CMC/Rh2 matrix at pH 5.8 is faster at the early stage, whereas it becomes slow at around 40 h. However, the release rate of Rh2 at pH 7.4 is relatively slow before 40 h, but it becomes faster after 40 h. It can be concluded that the ester bond between O-CMC and Rh2 can be easily broken in slightly acidic conditions, causing a large amount of Rh2 release at the initial stage under acidic conditions. 

Inflammatory pain, such as joint pain, burn-injury pain and neuropathic pain, is one of the most common types of chronic pain. Burning injury-induced inflammatory pain refers to the pain experienced as a result of a burn injury. When the skin is exposed to high temperatures, it can lead to tissue damage and trigger an inflammatory response in the body. This response involves the release of various chemical mediators, such as prostaglandins and cytokines, which contribute to the development of pain. The inflammatory process can lead to an increased sensitivity of the nerve fibers in the affected area, resulting in heightened pain sensations. This type of pain is often described as a burning or stinging sensation and can be accompanied by redness, swelling, and heat in the affected area. It is important to note that the management of burning injury-induced inflammatory pain typically involves a multidisciplinary approach. This may include the use of medications such as nonsteroidal anti-inflammatory drugs (NSAIDs) and opioid analgesics. Although opioids have good analgesic effects, they carry a significant risk of severe side effects. Thus, the development of more effective treatment methods is an important challenge and an urgent need in the field of pain research. Ginsenosides are steroid compounds and are mainly found in the traditional herbal plant ginseng (Panax ginseng C.A. Meyer). Ginsenoside Rh2, a major bioactive component of red ginseng extract, was reported to have various pharmacological effects including analgesic and anti-inflammatory effects. However, its low bioactivity and rapid degradation in living organisms has limited its widespread application. In this study, Rh2 was conjugated to O-CMC via ester bonding, extending the duration of pain relief, which significantly improved the current problems and provided future directions for the treatment of inflammatory pain. Indeed, in the current study, O-CMC/Rh2 showed good analgesic effects in the burin-jury induced inflammatory pain model. However, our study has some limitations; we were unable to target and deliver drugs to specific sites. Therefore, future studies should aim to develop a targeted and controlled-release drug delivery system for clinical applications. Our study provides valuable information for the preclinical and clinical studies of OS-CMC/Rh2 to treat chronic inflammatory pain.

## Data Availability

The data presented in this study are available on request.
